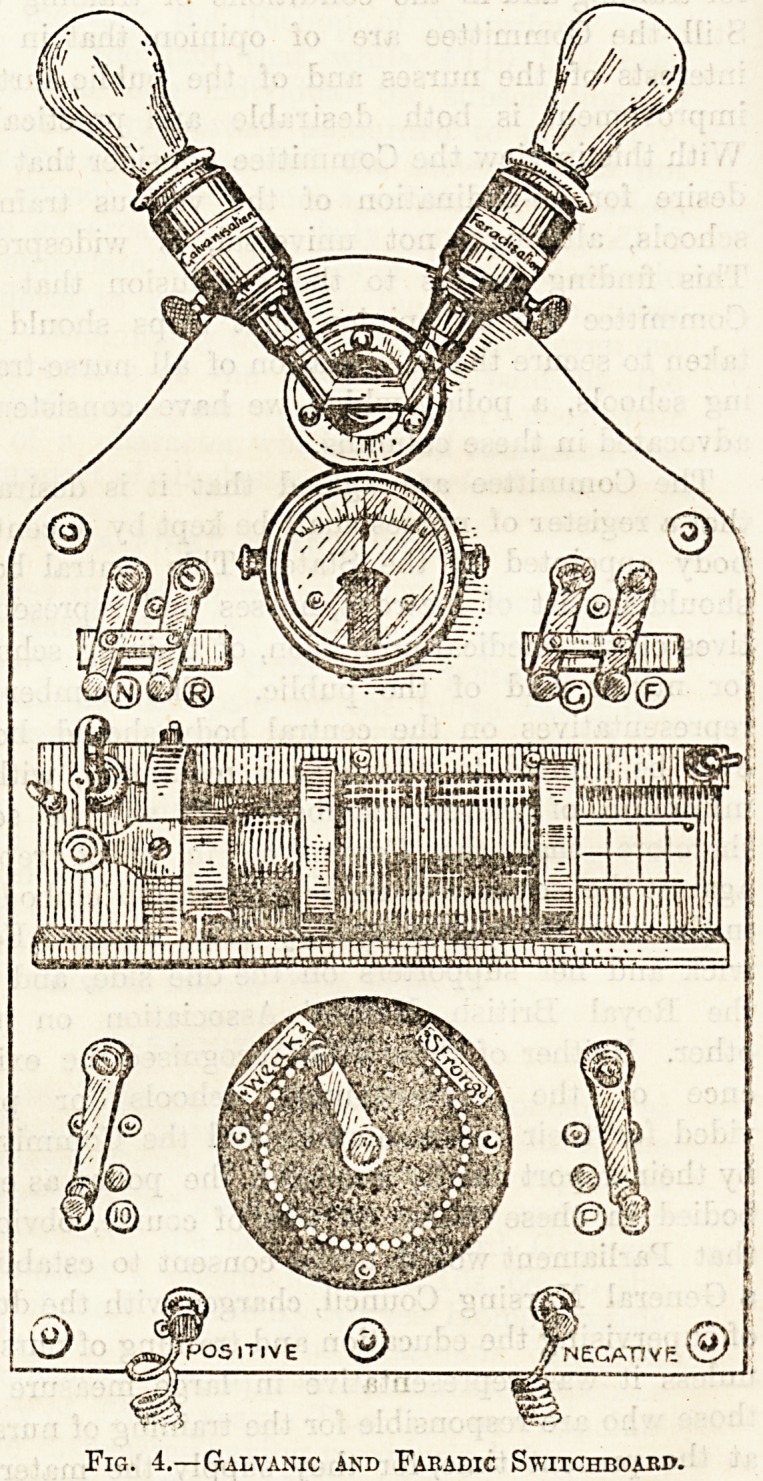# The Hospital. Nursing Section

**Published:** 1905-08-05

**Authors:** 


### The Hospital.

**Nursing Section.**

Contributions for this Section of "The Hospital" should be addressed to the Editor, "The Hospital" Nursing Section, 28 & 29 Southampton Street, Strand, London, W.C.

No. 984.—Vol. XXXVIII. SATURDAY, AUGUST 5, 1905.

### Notes on News from the Nursing World.

### THE ROYAL RED CROSS.

The King has conferred the decoration of the Royal Red Cross upon Miss Florence Ellen Adams-Williams, matron of Queen Alexandra's Imperial Military Nursing Service, in recognition of her special devotion and competency in the nursing of the sick and wounded of his Majesty's Army, and of her highly successful efforts in inaugurating the new nursing system at Netley. The decoration has also been bestowed by the King upon Mrs. Marion Jeffreys, in recognition of her services at the action of Bronkhorst Spruit in December 1880.

### THE DUKE OF CONNAUGHT'S TRIBUTE TO NURSES.

At the opening last week of the new Nurses' Home of the City of London Hospital for Diseases of the Chest the Duke of Connaught, having described the arrangements as very practical, simple, and suitable for the comfort and well-being of the nurses, said he hoped that the nurses would appreciate the change. He went on to observe that it was not many months since he had himself been in the hands of nurses, and he added that "he would always remember with gratitude the devotion with which they nursed him."

### ROYAL NATIONAL PENSION FUND FOR NURSES.

The *Glasgow Herald* of the 29th ultimo, in announcing the appointment of Sir John Ure Primrose, Bart., Lord Provost of Glasgow, as the representative of Scotch Nurses and Institutions on the Council of this Fund, has published a useful article on the Fund. The *Glasgow Herald* gives an outline of the history and aims of the Pension Fund, and sets forth the reasons why those Scotch nurses who desire to make provision against sickness and old age should join it. We have no doubt that the number of policies issued to Scotch nurses in the near future will show a considerable development.

### MR. LONG'S LEGACY.

The President of the Local Government Board stated the other day in the House of Commons that, after the report of the Departmental Committee on Nursing was issued, a considerable number of representatives were received by the department in connection with the recommendations of the Committee, and that an order dealing with the subject is "in preparation." We shall be glad to see that order. It was expected in vain while Mr. Long was at the Local Government Board, and he left the report as a legacy to Mr. Gerald Balfour, notwithstanding many assurances that it was intended to deal with the matter. Is it too much to hope that the order, to which Mr. Gerald Balfour refers, will be promulgated at any rate in November, which will be three years after the publication of the report of the Departmental Committee?

### OPENING OF THE NEWCASTLE-ON-TYNE HOME.

Commenting on the account given by our Commissioner of the nursing arrangements at the Royal Infirmary, Newcastle-on-Tyne, in July 1903, we expressed our surprise that the Newcastle people should have tolerated them so long. Under the conditions then prevailing, the minority of nurses who slept in the infirmary had to share rooms, three sometimes being in one apartment, while the majority who slept out had to walk to different parts of the city in the early morning or late in the evening, to the rooms provided for them. The matron herself described the quarters of those nurses who lived in the infirmary as "shockingly inadequate," and said that the only defence of the sleeping out business was that it was the best that could be made. All this, we are glad to state, has been put right at last—a year later than was expected—by the opening of the Nurses' Home in connection with the new Royal Victoria Infirmary, which is itself rapidly approaching completion. The new Home will accommodate 104 nurses, or between 30 and 40 more than the strength of the staff two years ago. It is a handsome, well-appointed, and commodious building, and it was warmly admired by the company who were present at the opening ceremony on Thursday last week. No complaint can in future be put forward respecting the housing of the Infirmary nurses, though, unfortunately, it will still be some time before full advantage can be taken of the Home.

### NURSES AND NERVOUSNESS.

During the hearing of a case in the Chancery division last week in which a resident at Hampstead sought for an injunction to prevent a house of which he is ground landlord from being used for the reception of patients attended by their own medical men, the plaintiff said that many complaints had been sent to him of nurses in uniform going in and out of the house very frequently. Asked whether he suggested that a nurse in uniform was such an offensive object that he could not himself bear to see one from his window, he replied that it was a great annoyance to him and to his family to see what was going on. Subsequently, he definitely stated that "seeing nurses about made his family nervous." We wonder how this family is composed. People who object to see funerals pass their window because they remind them of their own end are not uncommon, but it is a new idea to object to the sight of nurses in uniform because, we presume, they suggest the possibilities of sickness. We can only hope that if any member of this nervous family needs the attendance of a nurse she will be forthcoming.

### ABOLITION OF PRIVATE NURSING AT ROMSEY HOSPITAL.

At a special meeting of the subscribers to the Romsey Nursing Home and Cottage Hospital a proposal by the committee to alter the rules so as to do away with private nursing was discussed and agreed to. Hitherto the committee have supplied private nurses at terms varying from one to two guineas a week, but in future no nurse will be available outside the home for a longer period than one day or one night at a time, and this only with the idea of meeting immediate requirements, as a temporary measure, while the applicant is making arrangements to procure a permanent nurse elsewhere. It has been found that the maintenance of the private nursing branch has given rise to the idea that the institution is not a charity, and has tended to convey the impression that contributions are not needed. We have repeatedly pointed out that such combinations are inevitably open to misconstruction, and we have no doubt that the excellent charity at Romsey will benefit by the alteration in the rules.

### NURSES' SICK LEAVE.

The question of the duration of sick leave came before the St. George's-in-the-East Guardians the other day, owing to a ratepayer, who had not the courage of his, or her, convictions, protesting in an anonymous letter to the chairman against the salary of a nurse, off duty through illness, continuing to be paid to her. It transpired that the nurse in question contracted influenza while she was discharging her duties, and afterwards suffered from heart disease. The Guardians decided, as she had no relations who could keep her, to grant her three months' leave of absence, and afterwards extended it to six months. They have now decided that the salary shall be paid for another three months and that it shall then cease. We think that the Guardians have been very considerate in the matter, but we do not congratulate the ratepayer who objected to the nurse being granted sick leave.

### TERRIBLE BREAKDOWN OF A NURSE.

At Leeds, on Friday, a nurse of 46 years of age, who was charged with the wilful murder of her mother, was found guilty, but insane at the time the act was committed. The evidence went to show that the unfortunate woman, who had nursed her mother with great devotion, gave her laudanum because it was considered necessary to remove her to a mental asylum, had suffered serious breakdowns in health when she was sister and matron at hospitals in the north of England. These had occurred when she was subjected to unusually hard work, and they resulted in fits of morbid depression. There is no doubt that her mind had become unhinged; and the sad story gives point to the contention that none but women mentally and physically strong are qualified to undergo the strain which nursing in a position of responsibility involves.

### ESSEX GUARDIANS AND COTTAGE NURSING.

At a conference convened by the Essex County Cottage Nursing Association, at which delegates from boards of guardians were present, the nursing of persons receiving out-door relief and the question of the contributions by the guardians towards the support of nursing associations, were considered. Lady Rayleigh, who presided, said that owing to recent legislation the demand for district nurses was on the increase; and, eventually, a resolution to the effect that local associations affiliated with the Essex County Cottage Nursing Association should be encouraged throughout the county, and that contributions should be sought for from boards of guardians to this end. Copies of this resolution have since been sent to each guardian in the county. In taking it into consideration guardians will require, among other matters, to discuss the point how far the training undergone by the cottage nurses justifies them in availing themselves of the services of these nurses.

### MORE HONOURS FOR MISS PETER.

In addition to the honour paid to Miss Peter by the Queen, the retiring general superintendent of Queen Victoria's Jubilee Institute was presented last week by the Princess Louise with a laudatory resolution of the Council and a cheque. The Duchess of Buccleuch on the occasion mentioned the presentation made to Miss Peter by her Majesty, to which we referred last week.

### DUBLIN RED CROSS NURSING SISTERS' HOUSE.

At a meeting of the Managing Committee of the Dublin Red Cross Nursing Sisters' House last week, the secretary reported the death of the late lady superintendent of the Institution — Sister Alison—which took place since the last meeting of the committee. A resolution was adopted to the effect that the committee record their deep regret for the untimely death of Miss Alice Tenison Lyons, lady superintendent of the Institution from its foundation 23 years ago to the present, their high esteem of her many-sided abilities, combined with tact, prudence, and resource, which she displayed in the performance of her various duties, and they offer their sincere sympathy to her mourning relatives. It was announced that arrangements have been made, under the auspices of the committee, with the National Children's Hospital in Dublin and a large general hospital in England to give each probationer in the former institution a full three years' course of training.

### A POOR-LAW MATRON'S REPORT.

The matron of Brentford Poor-law Infirmary has issued her annual report. The training school has now been established for nine years, and it continues to make progress. Since July last year the three senior sisters left in order to take up other appointments, for private reasons, and two of the vacancies caused were filled by nurses trained at Brentford. There are now in the institution 25 staff nurses and probationers, as well as the sisters. We are glad to see that in her report the matron recognises the great pains taken by Miss Richford in teaching the junior probationers and coaching the seniors, so as to enable them to profit by the lectures given by the medical staff; the instruction afforded to the senior nurses in massage by Miss Brocks; and the teaching given in midwifery by Miss Mellor. At the final examination for the nurses in their third year, six presented themselves and all passed, each one of them obtaining above 75 per cent. of full marks. The names of the six nurses are—B. Taylor, C. Page, E. Palmer, E. Outram, C. Mann, and M. Mugglestone. It is a pleasing feature of Miss Moriarty's concise report that only one member of the nursing staff was off duty from illness during the year.

### A HOSPITAL "POUND DAY"

Pound Day at the Cottage Hospital, Kingsbridge, last year was an experiment; this year it was repeated almost "by request," and, now it is over, it promises to become a hardy annual. It is a useful and welcome mode of helping a local charity, and enables the poorest patients to help. It does not hurt anyone, for most of the "pounds" are bought at the shops; therefore the tradespeople do not suffer, and it affords every one the pleasant feeling of having given something. Of course it makes a deal of work, but the excitement of the long stream of pounds and the gratitude of former patients are ample compensation for the trouble. At Kingsbridge it was announced that pounds would be received from two to six o'clock on Tuesday, but they came for several days. In weight they were 428, and in donations over £7. One boy collected 18s. in the village near his home—the little Devonshire fishing village which is fast falling into the sea. There are now groceries, butter, coals, and eggs to last for months, and all were contributed with obvious pleasure.

### RESIGNATION OF A MATRON.

Totnes Cottage Hospital will shortly lose the services of the matron. The hospital has been established 20 years, and for upwards of 13 Mrs. Tucker has acted as nurse-matron. The committee, in announcing her resignation, testify to the excellent service she has done, and state that they appreciate the attention and skill, combined with good management, which have marked her duties during her long service. They have always, it is added, felt that under her control the household arrangements would be satisfactory, and that the patients would receive every attention and kindness.

### TRAINING AT GLASGOW MATERNITY HOSPITAL.

We understand that in September the Glasgow Maternity Hospital will have a few vacancies for nurses desiring to train. This is in consequence of the erection of the new hospital. The terms of admission are three months' training for £13 13s., four months for £15 15s., and six months for £21, with board and lodging. Nurses are advised to take at least four months' training, as otherwise they cannot qualify for the certificate of the Central Midwives Board, and those who have had no previous training in general nursing are required to do so. The Glasgow Maternity Hospital is the only maternity institution in Glasgow whose certificate is recognised by the Central Midwives Board.

### THE UNTRAINED MATRON AND THE TRAINED NURSE.

At the recent Northern Poor-law Conference Mr. W. M. Moorsom, Assistant-General Inspector under the Local Government Board, described the situation which arises in small workhouses when friction occurs between the matron and the head nurse. "The head nurse," he said, "comes to me and asks, with a very long face, if she can have a private interview, and for half an hour I have to listen to a list of the grievances and sins of the matron. Then I have to spend another half hour in listening to an equally terrible tale of the horrible sins of the head nurse." It is only fair to mention that Mr. Moorsom added, that lately in his district he has heard very little of this sort of thing, probably because the untrained matron has, in many cases, been superseded.

### A CONTRAST AT WATERFORD.

In moving the adoption of the report of the Waterford District Nursing Association at the annual meeting the Bishop of Waterford commented on the fact that while £110 had been received from one individual, the subscriptions of all the other supporters amounted to only £115. But for the £110, he added, the society would not be in existence. This is not a satisfactory position, and the generosity of the lady contributing £110 should stimulate the collectors of the association in their efforts to augment the income from other sources. Judging by the statement that eleven out of every twelve of the 7,404 visits paid by the nurses during the year were to the very poor, their work is of a character which should command the warm support of all classes in Waterford.

### A FIRST YEAR'S WORK.

The first annual report of the Tavistock District Nursing Association has been issued, and it is of a satisfactory character from every point of view. The number of visits paid by the nurse was 3,518, and the number of cases 151. The committee have a certain amount of money in hand from the Jubilee fund, but it is pointed out that subscriptions are needed in order to keep the undertaking going. A small sum was contributed during the year by the employés of the Gas Lighting Company. No doubt others will contribute if the committee take the necessary pains to induce them to do so.

### SHORT ITEMS.

Princess Christian attended a concert at Windlesham on Saturday afternoon in aid of the newly formed Nursing Association, and expressed during her visit her good wishes for its success.—The solicitors of the late Mr. Richard Grainger have forwarded a cheque for £151 16s. 6d. to the treasurer of the Wednesbury Nurses' Home towards the Endowment Fund.—The guardians of Acton Union Infirmary have appointed a female dispenser at a salary of £50 a year. This is a new appointment, the dispensing having previously being done by the medical officer.—Miss A. E. Ansdell and Miss L. Strickland have resigned their appointments as staff nurses in Queen Alexandra's Imperial Military Nursing Service.

### The Nursing Outlook.

"From magnanimity, all fear above;
From nobler recompense, above applause,
Which owes to man's short outlook all its charm."

### THE REGISTRATION OF NURSES.

After sitting through two sessions of Parliament the Select Committee on the Registration of Nurses has issued its report. It is not possible to be confident as to the exact bearings of this report until the whole of the evidence taken has been issued, together with the report of the proceedings at the meetings of the Committee itself. The report finds that there is a general opinion in favour of some change in the conditions under which nursing is now carried on in this country. The evidence shows that a considerable improvement has taken place of late both in the type of probationers entered for training and in the conditions of training too. Still the Committee are of opinion that in the interests of the nurses and of the public further improvement is both desirable and practicable. With this in view the Committee consider that the desire for co-ordination of the various training schools, although not universal, is widespread. This finding points to the conclusion that the Committee are of opinion that steps should be taken to secure the co-operation of all nurse-training schools, a policy which we have consistently advocated in these columns.

The Committee are agreed that it is desirable that a register of nurses shall be kept by a central body appointed by the State. This central body should consist of matrons, nurses and representatives of the medical profession, of training schools for nurses and of the public. The number of representatives on the central body should however be limited to fifteen as a maximum, with a minimum of eleven members. It will be seen therefore that the Committee in effect report against the scheme of nurse registration embodied in the two Bills promoted by Mrs. Bedford Fenwick and her supporters on the one side, and by the Royal British Nurses' Association on the other. Neither of these Bills recognised the existence of the nurse-training schools or provided for their representation, and the Committee by their report tacitly condemn the policy as embodied in these bills. It was, of course, obvious that Parliament would never consent to establish a General Nursing Council, charged with the duty of supervising the education and training of nurses, unless it was representative in large measure of those who are responsible for the training of nurses at the present time, for they supply the material and the means whereby that training is rendered possible. It is further satisfactory to note that the Committee condemn the advocates of a large registration fee, and that they consider that any such fee should not exceed one guinea in amount. It is further satisfactory that the Committee should recommend that the examinations should be held at and by the training schools, a policy which is the exact opposite to that embodied in each of the bills referred to. The Committee find that no evidence has been brought forward which substantiates a general charge of moral delinquency on the part of nurses. The Committee hold that whilst registration might prove a means towards checking some abuses, it could not discover and remove illegality, immorality and scandal on the part of a few of the least reputable women at present undertaking the duties of a nurse. So far as the existing nurses are concerned the finding of the Committee is that those who can produce satisfactory evidence as to efficiency and character should be placed on the register on payment of the registration fee. This finding enforces the practical usefulness of the scheme propounded by the Incorporated Society for the Higher Education of Nurses, and the sooner that body gets to work the easier will it be for Parliament to legislate in a practical manner on the subject of nurses' training and education.

The General Nursing Council should, in the Committee's opinion, decide what constitutes a recognised training school for nurses, having regard to the number of beds, the accommodation for probationers, the facilities afforded for learning, and the general standard and conduct of the examinations, for which latter purpose the Nursing Council should have the power of inspection. Providing all the interests involved are adequately represented upon the General Nursing Council this recommendation is likely to gain general acceptance. The Committee are impressed with the advisability of three years' training, though they are against the enforcement of a stereotyped rule, preferring that the matter should be left to the discretion of the Nursing Council when established by Act of Parliament. They recommend the annual publication of a register of nurses, and that the Nursing Council should have authority to amend the register and to exercise disciplinary powers for those registered nurses who may be guilty of serious misconduct in the discharge of their duty, or of moral delinquency.

It will thus be seen that the Committee, whilst recognising the impracticability of the schemes of registration so far submitted to Parliament, are nevertheless of opinion that it is desirable that a register of nurses should be kept by a representative Nursing Council appointed by the State, and with this in view it is desirable to bring about with all possible despatch the co-ordination of the various training schools throughout the country. We have not space this week to deal with the various aspects of the report or their bearing upon current nursing politics. All we have been able to do is to generally indicate the main findings of the Committee in regard to nurse registration only.

### Medical Electricity and Light Treatment.

By Kate Neale, Sister-in-Charge of the Actino-Therapeutic Department, Guy's Hospital.

### III.—GALVANISM AND FARADISM

(*continued from p.* 283).

### Testing Muscles.

It frequently happens that certain muscles and nerves in a limb are paralysed while others remain healthy, and it is a matter of great importance to ascertain accurately which are the ones affected. The investigation of this is spoken of as *Muscle Testing*. It consists of stimulating each nerve and muscle in turn, first with faradic and then with galvanic currents, and noting the result. A paralysed muscle, for example, fails to respond to faradism, when a healthy one would give a brisk contraction.

The examination is one that is made by the physician himself, as it necessitates an intimate knowledge of anatomy; but it would be well for you to understand the meanings of some of the expressions used in this connection. The exact area of skin over which it is necessary to apply the electrode to test any particular nerve or muscle is known as its "*motor point*"; the motor point, for example, of the biceps in the arm is about one-third the distance down from shoulder to elbow on the front of the limb.

When a healthy muscle is stimulated by galvanism at its motor point, it contracts more forcibly if the anode electrode be placed on it, and the current made (or closed) than when the anode is lifted off and the current broken (or opened). The former contraction is spoken of as the *anodal closing contraction*, or more shortly, *A.C.C.*, the latter as the *anodal opening contraction*, or *A.O.C.*

But a contraction stronger than either of these is obtained by pressing the kathode on the motor point (*kathodal closing contraction* or *K.C.C.*), while on lifting the kathode off the feeblest contraction of all occurs (*kathodal opening contraction* or *K.O.C.*). That is to say, in a healthy muscle—

K.C.C. is greater than A.C.C.
A.C.C. is greater than A.O.C.
A.O.C. is greater than K.O.C.

The change that usually takes place in a paralysed muscle is that A.C.C. becomes greater than K.C.C., and the muscle instead of contracting briskly reveals only a sluggish movement.

*Requisites for Muscle Testing.*

In preparing a patient for muscle testing by the doctor, you must first expose the limbs that require examination. When the legs are the affected part, the patient should lie on a couch, but this is unnecessary if the condition of the arms alone is to be investigated. In all cases, however, it is most important to place the patient in a good position as regards light, etc., that even the faintest contraction of a muscle may be readily detected.

Both galvanic and faradic batteries must be fitted up in readiness, but in place of the usual sponge electrode substitute one of prepared carbon covered with chamois leather. (Fig. 2b.) This electrode is commonly used in testing as it covers only a small area of skin, and the current that passes to the muscle beneath will be fairly concentrated.

Further, provide a basin of warm water for moistening the electrodes, and a piece of gutta-percha tissue to protect the clothes, or couch, from the indifferent electrode. This should be placed either at the neck, or, if the patient is lying down, beneath the lumbar spine. A towel, and writing materials for recording the results of the observation, will complete your preparations.

### Galvanic and Faradic Switchboard.

The facilities which most towns now possess for obtaining a constant supply of electricity from the mains has led in the past few years to a great increase in the number of installations, both public and private, where electro-medical treatment is practised on a large scale. More recently still, since the "Finsen" rays have proved themselves so valuable a therapeutic aid, many hospitals have

Fig. 4.—Galvanic and Faradic Switchboard.

appreciated the wisdom of organising well-equipped electrical departments where electricity in its various forms can be available for their patients. For this purpose the current required is obtained more satisfactorily from the electric lighting supply than from batteries, which even at the best are as liable to err as any human being. Cells are done away with, and the current from the main, weakened to a degree of safety for medical application, is led direct to the induction coil or galvanic terminals.

All the appliances requisite for galvanism and faradism are combined on one board—known as a *Switch-board.* The form of this varies according to the source of manufacture, but a convenient and efficient arrangement is seen in fig. 4, which represents the switch-board in use in the electrical department of Guy's Hospital. You will observe above, two electric lamps, the one labelled "Galvanism," the other "Faradism." They are interposed between the main and the terminals, and by the resistance they offer to the flow of electricity serve to dilute the current as it were to a safe extent before it reaches the patient. Immediately beneath these lamps is an ammeter (see page 239), the index of which registers the strength of current, while below on either side of this are two switches. The right-hand one is for altering the current from galvanic to faradic, and vice versa. When it is moved across to G, a galvanic current reaches the patient, but on switching it over to F this is changed to faradic. The other switch labelled N R is a reverser similar in its action to that described on page 255, and is used when the direction of the galvanic current is to be changed. In the centre of the board is an induction coil so devised that the current can be moderated by sliding the secondary coil away from the primary.

The strength of the galvanic current is varied by the collector seen at the lower part of the figure. The method of using this has already been described on page 255. Beneath this dial at the bottom are the two terminals, positive and negative, to which you attach the electrode wires, and flanking the dial on either side are two more switches of which the right hand one is in connection with the faradic coil. By switching it over to S the current to the patient is in the ordinary way from the secondary coil, but when at P it flows from the primary. The last switch (marked 1-10) in the lower left-hand corner has no effect whatever on either the galvanic or faradic current, but is connected with the ammeter alone, and is used in the following way. By pushing it over to "1" the index of the ammeter registers the exact number of milliampères that are passing, but when it is moved across to "10" the index marks only one-tenth of the real number. For example, if the switch were at "1," and the ammeter reading gave 20 milliampères, pushing it across to "10" would alter the reading to "2." This arrangement is necessary because the ammeter dial is graduated up to 20 only, and if a current of, let us say, 30 milliampères was passing the reading would necessarily still be only 20, but by moving the switch across to "10" the register would become 3 (*i.e.* 30).

### *How to Use the Switch-board.*

I. *For Galvanism.*—Attach the electrode wires to the screw terminals, and switch by F to G and N R to N. The terminal marked +, or positive, will now be the anode. Turn on the lamp engraved "Galvanism" and apply both electrodes to the patient. Then slowly move the handle of the collector round the dial until the ammeter registers the required strength of current. Administer treatment as directed. When the treatment is finished remember to turn off the collecting dial before lifting the active electrode from the patient. Lastly, switch off the lamp.

II. *For Faradism.*—Attach the wires as before. Move G F to F, and P S to S. Pull out the secondary coil as far as it will go. Turn on the lamp marked "Faradism." If the vibrator does not immediately act, start it by tapping lightly with the finger. Should the current be not strong enough, gradually push up the secondary coil over the primary. Apply the treatment ordered, and when completed switch off the light. There is no need to trouble about placing the electrodes in position before starting the current, nor in letting them remain until the lamp is turned off.

### The Nurses' Clinic.

### THE DISTRICT NURSE IN RELATION TO ELEMENTARY SCHOOLS. BY A SUPERINTENDENT OF DISTRICT NURSING.

The regular attendance of district nurses at elementary schools is desirable on the following grounds:—

1. The nurse has the opportunity of observing all the children of school age, instead of those only who form the families of her patients and who may chance to be at home during her visits. In addition to her own observation and inquiries she can be assisted by—

(*a*) The teachers, who will certainly have noted children short-sighted, deaf, ill-developed, unusually stupid, badly clothed, or in a dirty condition.

(*b*) By the parents, who, knowing that the nurse will visit the school, can instruct their children to seek her aid.

(*c*) By the elder brothers and sisters, who are often conscious of defects that busy, ignorant, or careless mothers have not perceived.

(*d*) By the children themselves, who will learn to apply to her—at any rate when in pain—and whose confidence can often be more easily gained by the nurse when they are at school than when in their own homes.

2. Many children of an age to do a little housework come to school with cuts and burns on their hands. These injuries have probably received no attention beyond having a piece of rag twisted round them; neglect which, in the most favourable cases, retards recovery and causes needless pain, while blood-poisoning is a not improbable result. Should the injury be of a serious nature, it will be easy for the nurse to ascertain whether it was purely accidental, or due to child's own carelessness, or wilfully inflicted, or due to the child's being given work unsuited to its age and strength, and she can then take any further steps that may be necessary.

3. Great expense to ratepayers and much suffering and loss of life would be spared, if the nurse had this opportunity of examining children and detecting those in early stages of measles, diphtheria, scarlatina, etc. It is difficult for educated people to believe how many children are at schoo who obviously ought to be in bed. For these children the nurse must search quite as zealously among the well-dressed

THE NURSES' CLINIC—*Continued.*

and well-cared-for as among the obviously neglected. When the average child would cry and beg leave to stay at home, the very poor one would go to school because it has nothing pleasanter to do, while the ambitious child of the clerk, or the thrifty artisan, will often conceal symptoms of illness from its parents from unwillingness to break its record of attendance. Medals for unfailing attendance have much to answer for, not only in causing epidemics, but in fostering a spirit of selfishness, and of placing the school before the home.

Untrained persons cannot realise how easy it sometimes is to detect infectious illnesses, and are apt to think that wholesale inspection of this kind would be purely perfunctory and lead to nothing but expense. A curious instance of its real efficacy came to my notice a few days ago. A doctor, who was inspecting a school in South London, picked out a boy recovering from a slight attack of scarlatina, and sent him home. In the afternoon he visited another school in the same district and sent home two little brothers who were sickening for the same complaint. The doctor did not know it, but these boys were first cousins, and lived in the same house.

4. Much can be done for children of deficient sight or hearing if the defects are discovered early in life, and their causes investigated, and the same may be said with regard to other malformations. A considerable number of the so-called stupid or inattentive children are suffering from remediable bodily defects.

5. The nurse can impress upon the children general rules for the care of teeth, and can see that hopelessly decayed first teeth, or first teeth that persist when the permanent ones are ready to come through, are removed. Many spoilt children among the poor are allowed to make themselves ill, and to disturb the household with their crying night after night, because none of their elders have the strength of mind to pull out a loose and perfectly useless tooth.

6. The nurse can carefully note the condition of the children's clothing, remembering that neat dresses often conceal thin rags held together with pins and twine, and that shabby and ugly ones not seldom cover stout and sufficient underclothing. It will be easy for an experienced nurse to ascertain whether bad clothing is due to the poverty, ignorance, neglect or cruelty of the parents.

7. The nurse can discover the children neglected in the matter of cleanliness, and can attend to them not only for their own sakes, but because it is a cruel injustice to decent parents to make their carefully-tended little ones sit next those literally swarming with vermin. School teachers have told me that the sole result of sending these children home to their parents with a message that they must be cleaned, is that the children play about the streets for a day or two and then return in precisely the same condition. The experienced nurse will, of course, readily distinguish between the superficial dirt of a child who has a bath and clean clothes once a week, and the settled blackness of the really neglected, and she will expect to find children at their cleanest on Monday morning and at their grimiest on Friday afternoon. She must also make allowance for local conditions. It is often impossible for the most careful country mothers to bathe their children regularly. A woman I know well was recently asked to go and do a day's charing. She would have been glad of the money, but she declined. "I've just done three weeks' washing, and there's such a lovely lot of rain-water still left that I want to stop at home and give all the children a good scrub down."

8. The nurse can pick out the ill-fed children and ascertain the cause of their sufferings. Poverty alone is responsible for very little of it. I recently visited a house where five stunted, apathetic children slept in one bed under one blanket. Their father had been in regular work for 23 years, and most of the neighbours on a smaller and less certain income lived not only in decency and comfort, but in positive refinement. I have often known well-paid artisans' children dirty, ragged, and ill-fed, and in some cases have found them thankfully accepting the charitable gifts of labourers who had far less than what is called "a living wage," but, nevertheless, had healthy, well-cared-for families, and food and clothes to spare.

The reasons why district nurses should be employed in this work in preference to nurses fresh from hospitals are :—

1. Their general knowledge of the home life of the poor sharpens their powers of observation, gives them a hundred clues to the meaning of what they see, and increases their resourcefulness.

2. They probably already know the parents of a considerable percentage of the children, and are known by repute to many more, and it will therefore be easier for them than it would be for a complete stranger to visit their homes (when necessary) and obtain favourable consideration of their suggestions for the children's good.

3. It is always advisable rather to widen the uses of a system already proved good, than to create a fresh and untried organisation.

### The Registration of Nurses: Report of the Select Committee.

The Select Committee appointed to consider the expediency of providing for the Registration of Nurses have agreed to the following report:—

1. The Select Committee on the Registration of Nurses was appointed in June 1904, and was re-appointed during the present Session of Parliament.

### The Witnesses.

2. Your Committee have examined 34 witnesses, among whom are included members of the medical profession, matrons of hospitals, superintendents of nursing institutions, nurses whose experience has been gained abroad as well as nurses who carry on their occupation in this country, a representative of the male nurses, representatives of various institutions and public bodies, including the Civil Service, besides ladies and gentlemen who are not professionally employed but who have given much time and work to the management of hospitals and asylums, and to the study of nursing questions both in the centres of population and in the rur[illegible] istricts.

3. Amidst many divergent views met with in this evidence there is a general opinion in favour of some change in the conditions under which nursing is carried on.

4. Your Committee have observed this tendency in the evidence of the medical profession and in that of the nurses themselves.

5. The evidence shows that a considerable improvement has taken place of late, both in the class of persons who undertake nursing and in the conditions under which they obtain their training and carry on their occupation.

6. It has been asserted in some quarters that registration is rendered requisite by reason of the amount of illegality, immorality, and scandal which at present continues undiscovered and unchecked. It is contended that registration would be an efficient instrument against these scandals, and would safeguard the public. In the judgment of your Committee, while registration might prove a means towards checking some abuses, no evidence which has been brought forward substantiates a general charge of moral delinquency.

7. On the other hand, there is a general concurrence of opinion that in the interests of the nurses and of the public further improvement is both desirable and practicable; and your Committee consider that the desire for co-ordination of the various training schools, although not universal, is widespread.

8. Upon the question of what changes in the conditions of nursing are desirable strong opinions are held and vigorous expression has been given to them.

9. In these circumstances unanimity could not be looked for.

10. The principal suggestions laid before the Committee are:—

(a) Registration of individual nurses.
(b) Registration of training schools for nurses.
(c) Licensing of nursing homes, institutions, and societies which supply or employ nurses.

Proposed Central Body.

11. Your Committee are agreed that it is desirable that a Register of Nurses should be kept by a Central Body appointed by the State, and that while it is not desirable to prohibit unregistered persons from nursing for gain no person should be entitled to assume the designation of "Registered Nurse" whose name is not upon the Register.

12. They recommend that this Central Body should be set up by Act of Parliament, and that its constitution should be defined in the Act, as was done in the case of the Central Midwives Board.

13. The Central Body should consist of matrons, nurses, and representatives of the medical profession, of training schools for nurses, and of the public.

14. Your Committee consider it desirable that the number of representatives should be kept within reasonable limits; they suggest 11 as a convenient number, and recommend that it should never exceed 15.

15. Your Committee recommend that the Central Body should admit to the Register of Nurses such nurses as have had a training at a recognised training school for nurses for a period to be determined by such body, and have satisfied their training school, whose certificate they must hold, stating that they are equipped with the knowledge and experience requisite for nursing, and that they are of good character.

16. They also recommend that the Central Body should decide what constitutes a recognised training school for nurses, taking into consideration the number of beds, the accommodation for probationers, the facilities afforded for learning, and the general standard and conduct of the examination; for this purpose the Central Body should have the power of inspection. Your Committee further recommend that the examination be held at and by the training school.

The Fee and the Training.

17. For the purpose of defraying the expenses of the Central Body a small registration fee should be charged. Your Committee consider that this fee should not exceed one guinea.

18. Your Committee have heard a large amount of evidence on the subject of the necessary period of training at a school. The great bulk of this evidence points to three years as the requisite period of training. They are themselves impressed with the advisability of such a period, but they recognise that a stereotyped rule might operate unfortunately. They therefore recommend that the minimum period should not be fixed by Act of Parliament, but should be left to the discretion of the Central Body.

19. There should be an annual publication of the Register of Nurses. For this purpose the Central Body should make provision for striking off the Register the names of those nurses who have died or who have ceased nursing, and also of those nurses who, in the opinion of the Central Body, have been guilty of serious misconduct in the discharge of their duty or of moral delinquency.

Existing Nurses.

20. With regard to existing nurses your Committee are of opinion that those who can produce evidence satisfactory to the Central Body both as regards efficiency and character should be placed upon the Register on payment of the registration fee.

21. The Committee are of opinion that it should be the duty of the Central Body at a date not later than four years after the passing of any Act for the Registration of Nurses to submit a Report to the Privy Council on the advisability of instituting a separate Register of nurses whose training is of a lower standard than that laid down for "Registered Nurses."

22. The claims for Registration of Mental or Asylum Nurses have been laid before your Committee. They are of opinion that a separate Register of "Registered Asylum Nurses" should be kept by the Central Body, to which should be admitted the names of nurses who have served for not less than three years (in not more than two Asylums), and have received the certificate of the Medico-Psychclogical Association, and can produce satisfactory certificates of good character.

Nursing Homes.

23. An analogous but separate question has come before your Committee, namely, the treatment of nursing homes and institutions. Nursing homes are deemed to include all homes and places conducted for profit where patients are taken in for treatment. By nursing institutions are meant those societies or bodies which supply nurses to the public.

24. The evidence laid before your Committee has led them to consider the licensing of such homes and institutions to be highly desirable. The licence should be issued by the county or county borough authority in whose area the home or institution is situated, and no such home or institution should receive the licence unless it is conducted in conformity with requirements to be laid down by that authority.

25. The county or county borough authority should be empowered to draft regulations to be approved by the Local Government Board, and to appoint inspectors who should have the right of entry and inspection.

26. It should be a condition of such licence that when a nurse employed in a nursing home or sent out by a nursing institution is not a "Registered Nurse" the fact shall be definitely stated.

### Central Midwives Board.

A meeting of the Central Midwives Board was held on Thursday, July 27th, and there were present the chairman (Dr. Champneys), Dr. Dakin, Mrs. Latter, Miss Paget, Sir William Sinclair, Miss Wilson, and Mr. Parker Young. Some letters from correspondents were read requiring elucidation of Rules E, 17 and 18, referring to notification by th midwife of puerperal fever and death.

With regard to the former point raised, the Board instructed the secretary to obtain further particulars of the position before giving an answer. The question as to death was whether, if the mother died when no doctor was present, though a doctor was in attendance, it was the midwife's duty to notify the case. Similarly in the case of a child feeble through premature birth, or of a still-born child. The Board held that in all these the midwife must notify.

A letter was read from the Clerk of the Gloucestershire County Council forwarding copy of a report of the Sanitary Committee urging an amendment of the rules with reference to the use of appliances and laying out of the dead. This report stated that the fact that all midwives had to carry appliances often led to serious misuse of them by those who did not understand them, and that it was extremely hard on midwives in country districts to be debarred from laying out the dead. The secretary was instructed to incorporate in his reply the fact that it was not in the power of the Board to alter the rules, and that with regard to the appliances they would suggest that the local supervising authority might have the midwives instructed in the proper use of them and the danger of their misuse.

A letter from the Secretary of the Midwives' Institute, forwarding a letter from Mrs. S. M. Budzanover, asking permission to undergo the Board's examination in the German language, was considered. The applicant had already passed the necessary German examinations, but was anxious to work in the East End. Her letter was written in very passable English, and the Board decided that they could not make an exception in her case.

The Secretary reported that at the recent examination there were 307 candidates, 223 in London, 31 in Bristol, 44 in Manchester, and 9 in Newcastle. The percentage of failures was 22·8. The expenses and receipts would, when all amounts were settled, nearly tally.

A report was read from the visitor at the examination at Bristol. Similar reports were submitted by Sir William Sinclair and Dr. Champneys, who acted as honorary visitors in Manchester and in London. There seemed to be a general consensus of opinion that the examinations were very satisfactory; the type of woman examined was good, and there was not much illiteracy in the papers. It appeared, however, that some of the teaching given to the candidates was not as complete and thorough as it might have been, and few of the candidates knew the rules as required by the Board. A vote of thanks, on the proposal of Mr. Parker Young, was unanimously passed to Dr. Champneys and Sir William Sinclair for their services.

The report of the Standing Committee was then received. The case of Ann Ashton, midwife, who did not summon a doctor till ten days after the confinement of the patient, was considered. The woman did not seem to have so acted through carelessness, but the patient died. The Committee recommended that she be severely reprimanded.

The application of Dr. J. H. Bellamy, of the Union Infirmary, Firvale, Sheffield, asking the Board to reconsider its refusal to recognise him as a teacher, was again considered, and further particulars having been obtained, it was decided to recognise him. A letter from Mr. Fowler, of Lewisham, asking the Board to reconsider its refusal to certify his wife as a midwife, was received. To protect themselves in future against these constant letters, the Board instructed the Secretary to bring no such applications for reconsideration before them unless some fresh facts were adduced. The case of Mrs. Fowler was not again considered.

It was decided to cite Maria Warr and Annie Broomhead, certified midwives, against whom *prima facie* cases had been established by the local supervising authorities for the city of Worcester and for Nottingham respectively.

A letter from Dr. Hugh Woods, general secretary of the London and County Medical Protection Society, Limited, calling attention to the conduct of a certified midwife as revealed by the evidence at an inquest, was deferred until further particulars could be obtained.

Applications for approval as an institution for the training of midwives and for approval as a teacher and as a certified midwife were considered.

The Board adopted the report of the committee.

On the motion of Miss Paget, it was resolved to send a copy of Section E of the rules free of charge to every woman receiving the Board's certificate.

### Victoria Park Hospital.

### OPENING OF THE NURSES' HOME.

On Wednesday last week the new home for the nurses at the Victoria Park Hospital was opened by the Duchess of Connaught. The Duchess was accompanied by the Duke of Connaught and Princess Patricia. Their Royal Highnesses arrived at the hospital at 4 P.M. precisely, where (by permission of the commanding officer, Lord Bingham, M.P.) a guard of honour of the London Rifle Brigade was mounted, and the band of the regiment played the National Anthem. Their Royal Highnesses were received by the treasurer, Sir Edward Sassoon, Bart., M.P., and Lady Sassoon; the chairman of the Committee of Management, Alderman Sir George Wyatt Truscott and Lady Truscott; the Lord Bishop of Stepney; and a reception committee, consisting of Dr. Heron, senior physician, and Mrs. Heron; Dr. E. Clifford Beale and Mrs. Beale; Dr. Vincent D. Harris; Dr. E. H. Colbeck, representing the Medical Committee; Sir M. M. Bhownaggree, K.C.I.E., M.P.; Mr. S. Forde Ridley, M.P.; Mr. Herbert Robertson, M.P.; Mr. G. H. Masterman, chairman of the House Committee; Mr. Godfrey W. P. Mellor, hon. solicitor; Mr. R. Langton Cole, F.R.I.B.A., architect: Dr. G. Basil Adams, resident medical officer; the senior officer in command of the Guard of Honour; Mr. H. Dudley Ryder, secretary; and Miss Clara C. Trafford, matron. The Duchess of Connaught was presented with a basket of flowers by a little patient (Ester Latliff). Their Royal Highnesses proceeded to the Nurses' Home, where the Bishop of Stepney conducted a brief religious service. A gold key was then presented to the Duchess by the architect, Mr. R. Langton Cole, for the purpose of formally opening the Nurses' Home.

The hospital was decorated with palms and flowers, and there were awnings in the garden. The nurses stood in file along the route from the hospital to the home. The Royal party had tea in the nurses' dining-room, after Sir Edward Sassoon had thanked the Duke of Connaught for his attendance and the latter had assured the company cf the pleasure which it gave the Duchess and himself to be present.

The home, which is built in the hospital grounds, is a long three-story building, consisting of 41 bedrooms, of which seven are at present unfurnished owing to lack of funds and nurses. With the exception of the matron, the whole of the nursing staff are now accommodated in the home. In each nurse's bedroom, besides the bed, is a suite of fumed oak furniture, consisting of a dressing table, washstand, and wardrobe, while the floor is covered with linoleum and a rug. The sisters' bedrooms are the same as the nurses', excepting that they are rather larger, and each has an easy chair and a table. Two bathrooms and lavatories are on each floor, and the stone staircases are covered with turkey carpet. At one end of the building there is an outside emergency staircase. The nurses' sitting-room, which is upholstered in red velvet pile, contains a grand piano, bookcases of fumed oak, many easy chairs, a Chesterfield couch, two writing-tables, etc. The walls are green, and the windows have green linen curtains. The sisters' sitting-room is similar. There is a tea-room, where the nurses can have tea when off duty, and a kitchen, fitted with a gas-stove, where they can boil water for themselves. The home is heated by hot-water pipes, and the ventilation is excellent. At present there are 26 nurses, but now that there is ample provision the authorities hope to increase the number to 32. The old quarters were very bad some of the cubicles being up in the roof of the hospital and without separate windows.

### Visit to the Spedale de Santa Maria della Scala, Siena.

BY A SPECIAL CORRESPONDENT.

This hospital was founded in the eleventh century by the canons of the Duomo, who then lived together like monks and were obliged to devote a part of their revenue to the assistance of the poor. In time it passed from their hands to those of the laity. For centuries it served as a lodging house for pilgrims, as well as an asylum for the sick and the poor. It was here that St. Catherine made her daily and nightly rounds amongst the ill and dying, resting in a cell adjacent to one of the chapels of the hospital during the intervals of caring for the sick. St. Bernardino, too, together with his companions, distinguished himself by his heroic care of the plague-stricken during the plague of 1400. It stands, a huge red-brick Gothic pile on the south-west of the Piazza del Duomo facing the beautiful cathedral. Seen from the Piazza the building looks solid and old, but dull, as there are few windows facing this way, and those high up. The nobly-proportioned Hall of Pilgrims, upon whose walls are frescoes concerning the history of the hospital painted by Domenico di Bartolo and Priamo della Quercia, from the single window of which a glorious view is obtained, together with two of the chapels, are shown to the public. A visit to these and the familiar smell of disinfectants made me long to see the whole building from a professional point of view, a wish which was shortly after gratified by the friendly aid of one of Siena's best and kindest surgeons, with whom the illness of a relative brought me in contact. Armed with an introduction from him to the Sister Superior, I sallied forth to look behind the scenes of a foreign hospital, trusting that my knowledge of French, sadly rusted during long years of hospital work, would enable me to make myself understood and to learn all I wished to know of the history and working of the hospital. Handing my introduction to the hall porter, I waited in the Hall of Pilgrims until a sweet-faced sister came along and conducted me through a maze of narrow and rather dark passages to the sisters' quarters, to which entrance was gained through a ocked door, keys to which hang from each sister's girdle. While waiting in the bare little visitors' room for the Sister Superior I was the object of much curiosity on the part of the sisters who passed the open door. They were evidently just returning from a service at the cathedral or chapel.

### A Quaint Costume.

There are only 24 all told, assisted by wards-men and women who come in by day or night. As the hospital has 400 beds, and every kind of case finds a bed beneath its roof, it does not seem as if the patients could possibly get the same care and attention they receive in our hospitals. The sisters wear a dark blue cloth habit, the wide sleeves shortened by being turned back, tight black woollen sleeves to the wrist keeping the arms warm, a blue linen apron with bib, and round the neck a large white linen collar with wide square ends reaching nearly to the waist. On the head a white cap, fitting close round the face; over that a second cap with quaint, wide-preading wings coming to a point in front, the wings turning up at the sides, the back pleated into a round like a Dutch bonnet. A girdle round the waist, to which various keys are attached, completes a quaint and picturesque costume.

### The Women's Ward.

The Sister Superior was not long in welcoming me; she has been at her post for 30 years and is deeply interested in the hospital. After a short conversation she brought in two French sisters, one of whom she requested to take me round, as she did not speak French very fluently. We first visited the women's wards, medical and surgical. The wards are long and lofty, but ill lighted—in most of them there is only one large window at the end — and according to English ideas the ventilation left much to be desired, and the space between the beds is much too small. The furniture is simple; small iron stands with three shelves at each bedside, a table, and a few benches for those who are up is all there is in most wards. A small glass cabin like a signalman's box is to be found in one of the wards in each section, and in this the sister sits and keeps her books. All clothing is provided; the men when up wear a long white flannel coat (it also does duty in bed), white cotton trousers, slippers, and a round white flannel cap. The women wear the same white coats with the necessary petticoats—even the babies are similarly attired. I saw one little mite looking very happy with a tracheotomy tube, which was inserted 5 months ago just in time to save his life. One woman was very ill with pneumonia (she had had her appendix removed quite recently); she sat propped up with pillows. A couple of screens and a sheet making a tent, and the "steam kettle"—a pot large enough to contain a good-sized round of beef—was steaming away on a gas ring. The beds are new, good spring mattresses, a nice height, and with no unnecessary ornamentation. The women's observation ward was once a chapel, and though the walls and ceiling are covered with beautiful frescoes, there is only one small window, very high up, and it is a very dull place; happily they only stay there for a short time after admission until it is decided into which ward they shall go.

### The Theatre and Kitchen.

The theatre, after the elaborate, not to say extravagant, examples one sees, looked very bare, but an attempt has been made to make it come up to present-day ideals. It is entered through sliding glass-doors, the floor is of mosaic with a drain for water, the walls are tiled half-way, the rest colour-washed, and it is lighted by one large window, three wooden operating-tables covered with blankets and mackintosh, some enamelled iron stools, and an instrument-case completed the furniture. In an anteroom at one side there are sterilizers for dressings, instruments, and water, and in the room at the other side were the wash-basins and an array of white coats and canvas shoes worn by all who enter the theatre during an operation. There is a man in charge of the theatre; the sisters attend some of the operations on the women, not all, as it is not always "convenable"; and, of course, they are never present when anything is done for the men. We looked into the kitchen with its fascinating array of copper-vessels, where the sister sat in her little box, and the men-cooks were busy preparing the evening meal, which is served at 4.30 p.m.

### The Men's Wards.

Crossing the Hall of Pilgrims one comes to the men's medical and surgical wards, long, lofty rooms containing either 24 or 30 beds, with one large window at the end. Here the lack of ventilation was even more apparent. There are no stoves or fireplaces. Hot air is admitted through gratings in the wall, and in most of the wards there was the metal basket, "scaldino," filled with hot wood ashes, over which the Italian tries to keep himself warm on those days when the sun, on which he so much depends, hides his face. There was a greater air of disorder and untidiness in these wards, due, I expect, to the fact that the sisters are not so much there, the male attendants looking after wards and patients. The out-patient department is on the floor below. We were unable to see the electrical department, as the man in charge was absent. Down more stairs and along a maze of dark, underground passages, once the pathological department, now happily removed to another building, and we came to the laundry, where my guide showed me with great pride the calender, steam washer, and wringer, this being the department under her charge.

Sorting Soiled Dressings.

In a dark little room I caught sight of a woman sorting soiled dressings, using her bare hands for the purpose. I inquired what it meant. "She is taking the gauze from the wool," was the reply, "the wool is then burnt, but the gauze is washed, boiled, and disinfected and used again for dressing." Does this apply to dressings from foul as well as clean wounds? "Oh yes, every dressing without exception is treated in the same way." The woman is supposed to apply an antiseptic ointment to her hands before touching the dressings. I wondered how our surgeons and sisters would appreciate such economy. On leaving the laundry we passed a large room where two men were busy doing up mattresses. As soon as a patient is discharged his mattress of cotton is disinfected, cleaned, and put into a clean cover. In the linen-room a sister and 12 women and girls were busy making and repairing the linen, everything being marked with the emblem of the hospital (the ladder of three rungs surmounted by a cross). The same quaint sign is found on many houses in the city, which I suppose are, or were once, the property of the hospital. At one time its revenues were considerable, but now it has the usual complaint of "bad times." Every patient who can afford it must pay 2 lire (about 1s. 6d.) a day, the commune or district paying for those unable to do so.

### My "Daddies."

BY THE MATRON OF A SUSSEX HOSPITAL.

We call them all "Daddies"—the old men—and I think they like it; it makes them think of their home, and they consider it a term of affection and tenderness. One dear old Daddy we had in our little hospital was quaint in the extreme, with rough country manners, but he was a good sort all through. He generally began his sentences with a shake of the head and "dang it all." The chaplain thought at first he was swearing and looked very serious; but no such thing, it was his favourite expression, to emphasise what he was saying. He took a great fancy to my sister (a nurse) who was with me for a short time, and after her departure remarked in his knowing way, "You have lost a good horse out of the team now, Matron"; and at another time, "How nice your little sister did look to be sure, she don't put her things on with a prong"; this struck me as a most unusual remark, but I hear it is a Sussex expression. After his broken leg was well he came to see me from time to time, and one day, when hobbling along in front of me, he suddenly turned round and said, "Dang it all, Matron, if I don't just like you; you are so homely like." Another "Daddy" was always so concerned because we were so busy. "Can't you sit down, Matron," he would say; and when one day the house surgeon threw himself into a chair near him exclaiming, "Oh! Daddy, I am so tired," his ready answer was, "And so be them women, I should think" (meaning me and the nurses).

One more "Daddy" I must mention: he was a Crimean veteran, and never tired of talking of his experiences; even in the dead of night he would be telling one of his many tales to the night nurse, and there was no getting away when once he began. He loved his pipe, and liked to get the nurses to fill it for him. I answered his bell one day, and asked, "'What do you want, Daddy?" "I'm sorry to trouble you, Matron," he said, "I thought one of them gels would light my pipe for me." One nurse was an especial favourite. "I think you must have a chap," he said to her, "for you know how to fill pipes so well." (Daddy was right. She has now married that "chap.") Poor old "Daddy"; he has since passed away at the age of 93. One of his greatest pleasures was to come and see his "nurses" after he left the hospital.

### Nurses on Holiday: A Visit to Vesuvius.

We had spent the morning at Pompeii, and then decided to visit Vesuvius to complete our impression of the power of the crater and the awful tragedy of "The Dead City." Three of our party, having had lunch at the native café, started to drive. They passed *en route* through typical Italian villages where the industry appeared to be macaroni-making and coral-working, the macaroni being hung on bamboo poles placed on the housetops to dry. The coral-workers were women, sitting at their drilling machines just in or outside the doorways. The drive was long and steep, the road partly paved with lava and hard cinder, the lava assuming grotesque shapes by the wayside. We must have looked very English, for a native band struck up the "National Anthem" followed by "Two Little Girls in Blue." We crossed over the new electric railway to the station of the funicula, boarding a car where several others were seated, and were drawn at a quick pace by a cable, trying hard not to think of what would happen if the rope broke. At the top of the line we had half an hour's walk through fine ashes, which were warm, and into which we sank almost knee-deep. The choice of a carrying-chair or a man and rope to pull us up was offered. We indignantly refused both, but finally gave in and availed ourselves of the latter, the former looking too unsafe at shoulder height. As we ascended the cone we heard rumblings and saw smoke, and felt also a slight vibration. At the top was a huge cavity, basin like, through a large hole in the bottom of which flame, lava and smoke escaped. I lay a few minutes peering over the edge and tried to take snapshots, when suddenly there was a deep rumbling and clouds of smoke, flame, and huge blocks of stone were shot into the air. This was repeated four times, when the guides hurried us away, saying the wind had changed and it was dangerous. Arriving at comparative safety, we gave coins to boys to have them burnt in lava, the smallest coins costing a franc each. The profit seems small enough for so dangerous a task, for the lava must be boiling to adhere to the coin. We again boarded the car and were thankful to be away. The thought of the awful tragedy, the swiftness of the burning death and the horror of it, played an important part in making the scene depressing and impressive, though interesting enough to make up for burnt hands and faces and ruined shoes. When one passes the villages on the way down one wonders how the inhabitants can be happy so near to danger, but the villagers love "their mountain," as they call it, and are not happy away from it.

### To Nurses.

**We invite contributions from any of our readers, and shall be glad to pay for "Notes on News from the Nursing World," or for articles describing nursing experiences at home or abroad dealing with any nursing question from an original point of view, according to length. The minimum payment is 5s. Contributions on topical subjects are specially welcome. Notices of appointments, letters, entertainments, presentations, and deaths are not paid for, but we are always glad to receive them. All rejected manuscripts are returned in due course, and all payments for manuscripts used are made as early as possible after the beginning of each quarter.**

### New Books for Nurses.

A Practical Guide to Cookery in West Africa and the Tropics. By Sister Cockburn. (London: The Scientific Press. Price 3s. net.)

The authoress in the preface to her book explains the reason which induced her to publish it. She says: "My sole object has been to provide a handy, practical, and useful book which may be referred to, the recipes being easily prepared, and likely to be acceptable as providing a little pleasant change in the rather limited menu of the ordinary West African cuisine, particularly in the case of invalids and convalescents." Sister Cockburn has not only succeeded in her object as far as those in West Africa and the tropics are concerned, but she has also compiled a very excellent collection of recipes, which will be useful in any English-speaking household at home or abroad. The special recipes for dishes available only in the tropics are not to be found in a general collection of recipes, as far as we know, and this should render the book welcome to those who have to superintend the kitchen in the localities for which it is especially intended. The excellent recipes for pilau and pooloot are likely to tempt the English housekeeper to experiment. It is possible to procure mangoes in England now, and so the recipe for that excellent dish pilau is quite easy to follow. Sister Cockburn concludes her book with instructions on invalid drinks and a chapter of useful nursing hints.

Essentials of Domestic Hygiene. By Clare Goslett. (London: Allman and Son, Limited.)

Mrs. Clare Goslett has collected in her book a large amount of practical information which should be very useful to householders and district nurses. If the chapters dealing with construction contain what seems almost a council of perfection to those who are powerless to alter their abodes to meet the majority of her suggestions, they include invaluable advice for those who contemplate building. No one should leave it to architect and builder to provide all those safeguards to health which modern sanitation indicates, but if supervision is exercised a work like Mrs. Goslett's should be consulted. We find in it all the practical information to guide us in the maintenance of a healthy home. Very wisely the various methods adopted for ventilation are discussed on their merits, and therefore the verdict in favour of the simple system of open window and fireplace becomes all the more convincing. This is one instance of the many which show that Mrs. Goslett has both a theoretical and a practical knowledge of her subject.

The "Battle of Life" Series of Health Tracts. By Mrs. Clare Goslett. (Price 1d. each.)

This series forms a most useful collection of health tracts, which will commend themselves both to district nurses and to visitors. Mrs. Goslett commences by treating of the enemies of health, and she next deals with essentials to health—air and sunlight, leaflets on rubbish, consumption, tinned foods, children, food and feeding, the care of teeth, skin, and the mind. The teaching in these leaflets is simple, practical, and practicable, the last being a very important point in reaching the poor. An instance of this is apparent in the leaflet on tinned foods. Mrs. Goslett points out the dangers and how to avoid them, but she makes no attempt to banish them entirely, because doubtless she knows that such action would be ineffectual.

Hints on the Care of Children. By Robert J. Blackham, Captain, Royal Army Medical Corps. (Southampton: W. Hobbs and Son. Price 4d.)

Taking as his text the words of Dr. Sims Wallace, "The feeding of children is the most important of all questions affecting the physical well-being of humanity," Captain Blackham has prepared a practical and simple little work for use in the married quarters of garrisons. It is full of tersely-worded information, which would be most useful to all mothers, both rich and poor. It is a little pamphlet which might well be introduced into the homes visited by the district nurse. The instructions are explicit and most complete, and within the limit of the intelligence of the least educated. There is a diet table for babies, one for older children, and one concerning the cutting of teeth. Clothing, bedding, and exercise are also discussed.

How to Cook for the Sick and Convalescent. By Helena V. Sachse. (Lippincott. 6s.).

There is one notable addition to the third edition of "How to Cook for the Sick." A chapter has been added treating entirely of substitutes for cane-sugar. And the author mentions her indebtedness to Dr. Judson Daland for the valuable suggestions on this point which resulted in the various recipes in which soluble saccharine with or without glycerine is employed for those who, though invalided, still possess a "sweet tooth." The making of Kumiss has also been elaborated, and a new way of albuminising foods, especially in a concentrated form, is explained. The rest of the book is full of excellent recipes, and it is impossible to find a better gift for a private nurse.

A Short Practice of Midwifery for Nurses. By Henry Jellett, M.D., F.R.C.P.I., L.M. New Edition. (London: J. and A. Churchill. 1905. 6s. 6d.).

This midwifery book is based upon the treatment adopted in the Rotunda Hospital, Dublin, and the present edition has been revised to make it simpler and more complete; many new illustrations, and a glossary of medical terms have also been added. Its object is not only to enable the nurse or midwife to deliver a case of normal labour, but also to enable her to know when to send for medical assistance, what to do until the doctor arrives, and how to give intelligent aid to the doctor.

### Everybody's Opinion.

### THE UNTRAINED NURSE AS MIDWIFERY PUPIL.

"An Old Pupil" writes with reference to "L.R.C.P.'s" letter: I cannot understand the late pupil's conduct. Probably it is owing to the late pupil being an uneducated and untrained woman, otherwise she would be better acquainted with the laws of professional etiquette and not poach on future pupils' preserves, but would go further afield for a practice, since obtaining that much-sought for and in the end gained L.O.S. certificate. Surely this is a most ungrateful attitude to take towards the teacher. "L.R.C.P." is a most excellent and clever instructor in midwifery, and takes the keenest interest in the coaching of his pupils, all of whom, with the exception of the late pupil, have successfully passed the L.O.S. examination the first time."

### INSPECTORS UNDER THE MIDWIVES ACT.

"Registered L.O.S." writes: Some time ago there was a discussion in your paper on midwives being made inspectors, and it was said that the posts were generally given to doctors. Now I learn on good authority that the inspector for my district is neither a midwife nor a doctor, but a lady guardian. On my arrival in this part of Kent I was told that the inspector had passed the bags. I will describe two. Both were made of cane open-work, one containing a certain compound and a douche can, with piping and glass vaginal tubes, a piece of boric lint, a nail brush, soap in box, and Higginson's syringe; the second a roll of strapping, a second tube and boric ointment. The bags were not dust proof, neither were there any linings. All other things which a midwife should carry, beyond a thermometer, were absent.

Upon making inquiries I was told by a doctor that it was "peculiar to the Act." I may also say that in my last parish the medical officer of health was inspector, but I never saw him, although he was appointed 12 months previous to my leaving. I do not mind a medical inspector, but I certainly do object to a person who neither understands nursing nor midwifery, to say nothing of surgery. In my parish there are midwives — not trained nurses — who go in the season gathering hops. In consequence their hands are very soiled, yet they attend midwifery cases. I should like to know if this is the "light or other employment" proposed in preference to general nursing.

### NURSING THE SCHOOLBOY.

"A. A. R." writes: Like "J. C." I have been interested in reading the article on "Nursing the Schoolboy," and quite agree with her that a sick-ward should be cheerful and bright. My sick-ward is at the top of the college, and we get too much sun, if that could be possible. Being at the end of the building, whichever of the three sides we look out of we see nothing but what is bright and pleasant. Even the boys often remark how pretty the scenery is. All visitors say that it is a charming spot. We can see all that is going on in the front and back fields. The day room is nearly 20 feet square, and the floor is covered with linoleum. It has three couches, chairs, and tables, and two windows. The sick-ward or dormitory opens out of the day room, and is half as large again. There is a large square of carpet in front of the fireplace, and at the end of the ward there is a large window. I generally manage to keep a few flowers and plants in the ward, and, as a boy remarked once, "It does look a bit like home!" A small room is partitioned off from the dormitory for the sick-ward maid, and if the boys are not ill enough to be watched all night there is always some one near, my bedroom being next to the day room. We have a sanatorium in the grounds for infectious cases, and I must own that the inside of it is far from being cheerful; but the outlook is lively enough at all times. I wish "J. C." had a little more brightness in respect to her surroundings.

### LADY DOCTORS AND MIDWIVES.

"J. W." writes: I am a district midwife in London, and have for some time past wondered if there are any midwives who will endorse the complaint I have to make. My grievance is that the lady doctor sent out from the hospital for 5s. is doing us a great deal of harm. Every one, I think, knows that our fees are low, and since the passing of the Act we are obliged to pay another visit, though we cannot claim a penny more; and now we have to fight against the 5s. fee of the lady doctor from hospital. When the poor can get their attendance for 5s. they will not pay the midwife's fee, and how are we to live if this goes on? Why do not these ladies charge the full midwife's fee if they want the work for experience, and give us all a chance? Only this week a patient came to me, and said "I have found out that the lady doctor goes out for 5s. I am going to have her, so I cancel my engagement with you." I hope some one else will give their views on the matter. The life of a midwife need not be envied; she is always tied to her work, scarcely ever having any change or pleasure, and it is hard that the lady doctor should be allowed to cut the ground from under our feet.

∴ It appears from inquiries which we have made that the hospital in question does impose a charge of five shillings for lying-in cases. But the curious ground upon which this fee is required, is that the institution is not a free hospital. We may have more to say on the matter presently.—Ed. The Hospital.

### NURSING THE HOP-PICKERS.

The Rev. Francis G. Oliphant, Rector of Teston, Honorary Secretary of the Church of England Mission to Hop-pickers, writes: May I appeal through your columns for lady workers among the immigrant hop-pickers in the coming season. We want 20 nurses, and over 30 lady helpers. We have obtained some of these, but need more. We pay board and lodging and travelling expenses, but give no remuneration. We undoubtedly see great results from our past efforts in this direction, and the self-denying work of the ladies is greatly appreciated by the pickers. The nurses work in temporary hospitals, chiefly for children, dressing wounds, or visit in the different encampments the sick cases. The medical men are most thankful for the help given. Too often the nearest hospital or infirmary ward is five to eight miles distant, and when, as in some cases, there is a sudden influx of 4,000 pickers in a parish, the medical assistance obtainable is quite incapable of coping with the numerous cuts, chiefly from tins, poisoned wounds, and burns, etc., suffered by our visitors, and a nurse's assistance is most invaluable; in many cases a nurse who can cycle adds greatly to her value in such a position, being able to go from encampment to encampment in much less time than would be otherwise necessary. May I ask any of your readers willing to undertake this self-sacrificing, but most beneficent and helpful work, to communicate at once with Miss Harvey, 265 Vauxhall Bridge Road, London, who kindly undertakes this part of our work, as hop-picking will probably commence the last week in August. Gifts of old linen and illustrated papers can be sent to me here, either by post or by rail, to Wateringbury, South-Eastern and Chatham Railway.

### PRIVATE NURSES AND THE SERVANT QUESTION.

"Sister Gertrude" writes: The experience of an old nurse who has done private nursing for many years may be interesting to a young nurse just leaving hospital and going out into the world. After the regular routine of ward work, and being used to acting under orders of staff nurse or the sister, there is a certain amount of freedom, but there is also a great amount of responsibility. The well-trained nurse may be equal to it, but she stands alone and must often decide and act quickly. I shall never forget my first bad case. The doctor gone, the family all tucked in their bed, and these words ringing in my ears—"It's all right, we have a nurse in the house; so we can all sleep." The patient delirious, life just hanging in the balance, an awful thunderstorm going on by way of an extra, and the doctor six miles away! The general difficulty in private nursing is the servant question. Servants dislike a nurse. She makes extra work, I am afraid often somewhat unnecessarily, and treats the maids as persons beneath her, whilst the servants in return argue that nurses are paid for their work and are no better than they are. I find that to treat the maids with due respect, give as little trouble as possible and still hold your own, is the happiest way out of the difficulty. I had recently rather a funny experience. My patient went away for a change and left me in charge. When the mistress is away the maids like play. So far, so good. But when visitors are invited, suppers given, and late hours are kept, it strikes me as being more than play. This happened. Then came the question, "Ought I to tell?" As a rule, "Nurse" sees as little as possible, but when she is positively asked she has to decide what to do. I thought it well over, and decided to warn the principal offender and explain the wrongdoing, recommending her to give up such ways, ending with this remark, "We nurses do not go into houses to make mischief." This was all settled then, as I thought; but suddenly the girl came back to my room. "Nurse, I cannot understand how you can be a friend to me, who am a servant, and to your patient, my mistress, because the Bible says 'Ye cannot serve God and Mammon.'" I didn't ask which was which.

### Appointments.

Ayr District Asylum.—Miss L. Lockerbie has been appointed assistant matron. She was trained at the Western Infirmary, Glasgow, and has since done private nursing in Edinburgh.

Berkshire County Nursing Association.—Miss Constance Perceival has been appointed superintendent. She was trained at the Royal Infirmary, Sheffield, and has since been district nurse in Shoreditch and Paddington. She is registered under the Central Midwives Board.

Chelmsford Union Infirmary.—Miss Florence Whitren has been appointed charge nurse. She was trained at the Incorporation Infirmary, Shirley Warren, Southampton.

Dudley Union Infirmary.—Miss M. Lanham has been appointed charge nurse. She was trained at the Incorporation Infirmary, Shirley Warren, Southampton.

Dunster and Minehead Village Hospital, Somerset.—Miss Annie Milner Carr has been appointed matron. She was trained at the Royal Hants County Hospital, Winchester, where she did sister's holiday duty. She has since been sister at the Royal Isle of Wight County Hospital, Ryde, and has assisted the matron in her duties.

Isolation Hospital, East Malling.—Miss Bertha Conroy has been appointed staff nurse. She was trained at Addenbrooke's Hospital, Cambridge, and has since been nurse at the Northampton Borough Hospital and the Maidstone Sanatorium.

Malton Cottage Hospital.—Miss Lilian Lloyd has been appointed matron. She was trained at the Jessop Hospital for Women, Sheffield, and the General Infirmary, Bolton. She has since been charge nurse at the Park Hospital, London, S.E., night superintendent at the Royal Infirmary, Halifax, and night superintendent at the Royal Hospital for Chest Diseases, City Road, London.

Nantwich Workhouse Hospital.—Miss Maud Mellor has been appointed superintendent nurse. She was trained at Monsall Fever Hospital, and has since been charge nurse at Nantwich Workhouse Hospital and superintendent nurse at the Wolstanton and Burslem Workhouse.

National Hospital, Queen Square, Bloomsbury.—Miss Annie Richards has been appointed staff nurse. She was trained at the Incorporation Infirmary, Shirley Warren, Southampton.

Portsmouth Workhouse Infirmary.—Miss Florence Alice Foyster has been appointed matron. She was trained at the Whitechapel Infirmary, where she has since been staff nurse, night superintendent, and midwife. She has also been first and second assistant matron at Poplar and Stepney Sick Asylum.

Royal London Ophthalmic Hospital.—Miss F. A. Haig Brown has been appointed lady superintendent and matron. She was trained at St. Thomas's Hospital, and has since been matron of the District Infirmary, Ashton-under-Lyne, and sister-in-charge of the Nightingale Home, St. Thomas's Hospital.

Royal Seamen's Hospital, Cardiff.—Miss Annie Barnes has been appointed charge nurse. She was trained at the Incorporation Infirmary, Shirley Warren, Southampton,

Shoreditch Infirmary.—Miss Mary Millington has been appointed sister. She was trained at the Blackburn and East Lancashire Infirmary. She has since been charge nurse at Peterborough Infirmary and Stroud Hospital; sister at the Victoria Infirmary, Glasgow; Army Reserve sister, South Africa; and lately she has been attached to the West of Scotland Electro-Medical Institute.

West Norfolk and Lynn Hospital, King's Lynn.—Miss Muriel Thomson has been appointed sister. She was trained at the Norfolk and Norwich Hospital. She has since done private nursing for the Bedford Nurses' Institute, and has been sister of the male medical ward at the Essex and Colchester Hospital.

Yokohama General Hospital.—Miss A. G. Peacock has been appointed sister. She was trained at the Royal Infirmary, Bradford, where she has since been sister. She has also done private nursing in England and Hongkong, and is registered under the Central Midwives Board.

### Needle Accidents.

BY AN IRISH NURSE.

Few people are aware of the number of accidents which occur owing to needles being left about. If I give some of many instances which I have come across, it will be easy to judge from the experience of one person only, what must be the annual number of " needle cases " ?

A mother while playing with her baby on her lap was alarmed at the sudden screams of the child, when on undressing it she found that a needle which she had stuck into the bodice of her dress had penetrated the baby's back, and it was only extracted with difficulty by a surgeon.

A young girl upset a case full of needles on the floor. Kneeling down to gather them up one or more got embedded in the knee. After many months' suffering an operation was performed, when many pieces of broken needles were found. But finally the leg had to be amputated.

A man on springing out of bed felt a sharp pain in one foot, not having any idea of the cause. On consulting a surgeon, with the aid of the Röntgen rays, part of a needle was discovered in the sole.

I myself once had a bad time owing to the point-half of a large needle having gone into the palm of my hand when I was hurriedly dusting. The muscles contracted, and for some time I could not open the hand. An operation was not advised. However, greatly to my relief, the needle came out at the wrist three months afterwards. Had the needle broken as it entered my flesh I might have lost the use of my hand.

I could relate other cases among children and babies, and of needles entering a woman's breast owing to their being stuck in the front of the dress. I think I have sufficiently shown the results of leaving needles about, or putting them in dangerous places—such as the dress, curtains, arms of chairs, or table-covers.

Nothing is easier made than a needle-case, and the cost—well, nothing; and this article, if always utilised, would save untold trouble and anxiety.

### Presentations.

Victoria Hospital, Keighley.—Miss E. C. Laurence, R.R.C, having resigned the matronship of the Victoria Hospital, Keighley, in order to take up some new work in London, was presented before leaving with a handsome travelling clock and a silver frame by the nursing staff, and with a lace-worked tablecloth by the servants.

Foundling Hospital Infirmary.—On the occasion of Miss Tawney resigning the post of infirmary superintendent at the Foundling Hospital, London, she was presented with a silver tea-service by some of the governors and members of the staff, with their best wishes for her prosperity in her new career as health visitor.

North Staffordshire Infirmary.—Prior to the departure of Miss H. L. Pearse, late superintendent of nurses at North Staffordshire Infirmary, Stoke-on-Trent, to take up her duties as matron of the Great Northern Central Hospital, London, she was presented by the nursing staff with a silver kettle and spirit-lamp, as a token of their esteem, and also with a gold chain from the late resident officers, a set of silver-mounted ebony brushes from the domestic staff; a small breakfast service, handsome large vase, and bouquet of flowers from other members of the staff.

### Where to Go.

The August Bank Holiday.—On Saturday the New Palace steamer *Koh-i-noor* will leave Old Swan Pier, London Bridge, at 8 a.m. for Margate, on Sunday, August 6, at 8.30 a.m., and on Monday, August 7, at 8 a.m., for Southend, Margate, Ramsgate, Deal, and Dover. There will also be an additional sailing by the *Koh-i-noor* on Tuesday, leaving Tilbury at 9.30 a.m., special train from Fenchurch Street at 8.27 a.m., St. Pancras at 8 a.m. for Southend and Margate.

### Notes and Queries.

**REGULATIONS.**

**The Editor is always willing to answer in this column, without any fee, all reasonable questions, as soon as possible.**

**But the following rules must be carefully observed.**

1. **Every communication must be accompanied by the name and address of the writer.**
2. **The question must always bear upon nursing, directly or indirectly.**

**If an answer is required by letter a fee of half-a-crown must be enclosed with the note containing the inquiry.**

*Freidenheim Hospital.*

(144) Can you tell me the address of the Freidenheim Hospital which, I believe is in the Islington district.—*Southport.*

The address of the Freidenheim Hospital is Upper Avenue Road, Swiss Cottage, N.W.

*Sanitation.*

(145) Will you kindly tell me what examinations are needed to get a certificate in sanitation and where I could apply for same?—*E. S.*

Write to the Sanitary Institute, Margaret Street, London, W.

*Seaweed.*

(146) Do you know where "Veno's Seaweed Tonic" is made, and, if so, will you kindly let me have the address? Could you also let me know of any people who would like to be supplied with violet leaves and edible seaweed, as I should be glad to hear of some. Could you advise me how to find someone to take these things?—*F. M. B.*

"Veno's Seaweed Tonic" is made by the Veno Drug Company, Manchester. You might write to them and also to Messrs. Burroughs, Wellcome and Co., Snow Hill Buildings, London, E.C.

*Books for Probationers.*

(147) Will you kindly tell me the best books to teach probationers from?—*M. C. B.*

"A Handbook for Nurses." By J. K. Watson, M.D. 5s. "Elementary Physiology for Nurses." By C. F. Marshall, M.D. 2s. "Elementary Anatomy and Surgery for Nurses." By W. M. Eccles, F.R.C.S. 2s. 6d. "Practical Guide to Surgical Bandaging and Dressing." By W. Johnston Smith, F.R.C.S. 2s. "Surgical Ward Work and Nursing." By Alexander Miles, M.D. 3s. 6d. These are all published by the Scientific Press, Limited, 28 and 29 Southampton Street, Strand, London, W.C.

*State Registration.*

(148) Will you kindly tell me, in the event of State registration for nurses coming into force, how do maternity nurses stand? Will they have to train in a general hospital for three years?—*Nurse Amy.*

We cannot anticipate State registration.

*Central Midwives Board Examination.*

(149) Can you tell me which are the best books to study for the Central Midwives Examination?—*C. M. P.*

"How to Become a Certified Midwife." By E. C. Appel, M.B., 2s., published by the Scientific Press, Limited, 28 and 29 Southampton Street, Strand, London, W.C., will give you all the information you require.

*Aix-La-Chapelle Treatment.*

(150) I shall feel most grateful if you can give me some information where I can obtain gratuitously the Aix-la-Chapelle treatment for an ovarian tumour to be treated by absorption?—*Nurse H.*

We do not know where you could obtain the treatment gratuitously, nor does the treatment you suggest seem suitable.

*Sanatoria.*

(151) I should feel obliged if you could give me the address of the Nordrach Sanatorium, and the Arosa Sanatorium, Upper Engadine, or any other sanatoria in Germany or Switzerland where English nurses are employed as I want to get work abroad for the winter?—*J. C.*

The address Nordrach Sanatorium (Dr. Walther), Black Forest, and Arosa Sanatorium, Upper Engadine, will suffice. You might also write to the Davos Home, Davos Dorf, Switzerland.

**Handbooks for Nurses.**

| | Post Free. | |
|---|---|---|
| "A Handbook for Nurses." (Dr. J. K. Watson.) ... | 5s. | 4d. |
| "Nurses' Pronouncing Dictionary of Medical Terms." ... | 2s. | 0d. |
| "Art of Massage." (Creighton Hale.) ... ... ... | 6s. | 0d. |
| "Surgical Bandaging and Dressings." (Johnson Smith.) | 2s. | 0d. |
| "Hints on Tropical Fevers." (Sister Pollard.) ... ... | 1s. | 8d. |

Of all booksellers or of The Scientific Press, Limited, 28 & 29 Southampton Street, Strand, London, W.C.

### For Reading to the Sick.

"A BROKEN AND A CONTRITE HEART."

Lord, when my heart was whole I kept it back,
And grudged to give it Thee.
Now then that it is broken, must I lack
Thy kind word "Give it me!"
Silence would be but just, and Thou art just.
Yet since I lie here shattered in the dust,
With still an eye to lift to Thee,
A broken heart to give,
I think that Thou wilt bid me live,
And answer "Give it me."

*Christina Rossetti.*

All the great moral as well as the religious reformations have been begun and carried to a successful end by suffering. We may, indeed, take up our cross and see in it nothing but an instrument of pain; we may writhe under its torture and fill our life with the selfish cry of our misery, saddening also the lives of those around us; or we may exalt it into a Divine messenger, bringing it daily into the full light of God's presence, and steeping it in His brightness; so shall it become luminous for the dark hours of our lonely suffering, and its blessed light shall point others to the Lamb of God Who taketh away the sin of the world.

Although David was "the man" whose "heart was perfect with the Lord his God," he was essentially human in all his feelings. He spake them out to God, he communed with him, and found a help and strength and sympathy which he never would have found in an unnatural repression or in an exaggerated stoicism of expression. It is communion with Christ which the soul requires which is buried with Him in the great baptism of suffering; if it has risen with its Lord, and has touched the unseen foundations, of necessity it must seek those things which are above.

Even so should the Christian rise from his time of trouble; whether it be a heart trouble which must leave a scar for ever, or some infirmity of body or estate, he should, if he wishes to be God's "fellow-worker," learn from his own past how to minister to those in sorrow. The knowledge of suffering, if thus learnt by the teaching of the Holy Spirit, brings with it a power which is almost limitless in its effects for good upon others.

What then are we as earnest Christians to do with our portion of suffering? If it is true that God sends us painful ordeals, not so much for the punishment of our sins as for the sake of purifying and elevating our aspirations, should we not look upon this discipline as the very highest glory which God can bestow on us here? The more keenly we feel the pain in body, soul, and spirit, so much the more are we being prepared for the glory which shall follow. "If we suffer, we shall also reign with Him."—*Mrs. C. Campbell.*

Prostrate your soul in penitential prayer!
Humble your heart beneath the mighty hand
Of God, Whose gracious guidance oft shall lead
Through sin and crime the changed and melted heart,
To sweet repentance and the sense of Him.

*Clough.*

Those who inflict must suffer, for they see
The work of their own hearts, and that must be
Our chastisement or recompense.

*Shelley.*

## Figures and Tables

**Fig. 4. f1:**